# The Behavioral Intention to Adopt Circular Economy-Based Digital Technology for Agricultural Waste Valorization

**DOI:** 10.3390/foods12122341

**Published:** 2023-06-11

**Authors:** Teerapong Pienwisetkaew, Sasichakorn Wongsaichia, Benyapa Pinyosap, Supakkarn Prasertsil, Kunjira Poonsakpaisarn, Chavis Ketkaew

**Affiliations:** 1 International College, Khon Kaen University, Khon Kaen 40002, Thailand; 2 Center for Sustainable Innovation and Society, Khon Kaen University, Khon Kaen 40002, Thailand; 3 Faculty of Business and Economics, University of Antwerp, 2000 Antwerpen, Belgium

**Keywords:** agricultural waste, circular economy, digital platform, user intention

## Abstract

Thailand generates considerable amounts of agricultural food waste. This research focuses on the manufacturing and retail agricultural food system in the northeastern region of Thailand. Our study aimed to investigate the user segments and factors that influence users’ behavioral intentions to utilize mobile technology for agricultural waste valorization. This study is based on the Unified Theory of the Adoption and Utilization of Technology (UTAUT2). In order to classify these segments, we performed a cluster analysis using demographic variables: gender, age, and income. In addition, the researchers employed a method known as multigroup structural equation modeling to determine and contrast the users’ behavioral intentions. The results showed two types of users: (1) older users with various income ranges, and (2) younger users with a low-income range. Explicitly, age and income were the significant variables for the demographic segmentation, but gender was not. The results also revealed that social influence, price value, and trust highly affected the behavioral intentions of older and various-income users, but did not influence younger and low-income users. However, privacy strongly affected the behavioral intentions in the younger segment, but not those in the older one. Lastly, habit or regularity influenced the behavioral intentions of users in both segments. This study highlights implications for how developers and practitioners might adapt their platform strategies using a circular agricultural platform and user behaviors.

## 1. Introduction

Food waste is produced in large quantities in industrial production, agricultural processing, domestic consumption, and retail. It is one of the world’s most significant problems in both wealthy and developing nations. Every year, over one billion tons of food is lost or thrown away, accounting for one third of all the food produced for human use, resulting in significant social, economic, and environmental problems [1]. In terms of the total municipal solid waste yearly (MSW) produced in ASEAN, Thailand has the second highest amount of MSW (26.77 million tonnes/year), while the first is from Indonesia, with 64 million tonnes/year, and in third place is Vietnam, with 22 million tonnes/year [2]. The food industry generates a large amount of by-products, which causes substantial economic, managerial, and environmental issues, in addition to a significant loss of precious resources. The fruit processing sector falls under the category of food processing. International law addresses the disposal of fruit industry by-products (peels, seeds, and oilseed meals) as a difficulty. Finding new prospective application areas for these wastes to further utilize them for the creation of high-value goods has therefore received increased attention. Currently, the fruit processing industry discards its by-products (peel and seed) because they have no economic use. Because of the scarcity of landfills and the high cost of transportation, the disposal of these wastes incurs extra costs for the fruit processing industry. Due to their high chemical oxygen demand (COD) and biological oxygen demand (BOD), as well as their rapid rate of disintegration, which provides an ideal environment for the reproduction of insects, they should be disposed of carefully.

Both manufacturers and retailers are likely to throw away unconsumed processed and fresh foods. This amount of waste from agricultural production, including the manufacturing and retail industries, is classified by a proportion of 50%, which is made up of raw and cooked peel and seed. According to the United Nations Food and Agriculture Organization, the breakdown of logistical and infrastructural systems causes some countries to waste 55 percent of their agricultural output [3]. Whether pickled, preserved, or dried, these processed products cause problems with waste disposal and affect pollution in terms of smells and community cleanliness, which cannot be dealt with efficiently [4,5]. Based on previous research [6,7,8,9], the development of agri-waste management platforms could still be novel, leaving a research gap in the development of digital platforms as innovative tools for agricultural waste management in Thailand.

Moreover, the circular economy is used to solve food waste, by converting this waste into high-value-added products [10]. Hence, developing agri-waste digital platforms to use as an intermediary for the purchase of scraps from fruit/vegetable processing can help to reduce the agricultural waste issues in northeastern Thailand. This northeast region contains 46% of the agricultural holdings in Thailand and 47% of its farm area, with an average holding size of 3.2 hectares. Wastes, in this case, may come from fruits such as mangoes, oranges, pomelos, coconuts, limes, corns, and sugarcanes, and vegetables such as tomatoes, lettuces, kales, and cabbages. Farmers or sellers play a significant role in the platform by bringing the waste to be sold using this platform. Still, most of the previous studies on digital platforms as an innovative tool for managing food waste have focused on technology and innovation to develop digital platforms, leaving a second research gap in exploring behavioral intentions in a deeper dimension with regard to digital platforms [11,12,13]. Previous research related to circular-economy-based digital technology has included process-based information systems (PBIS), which help with digital tracking, modeling, and sensing for recycling, reuse, and remanufacturing [14,15].

**Figure 1:**
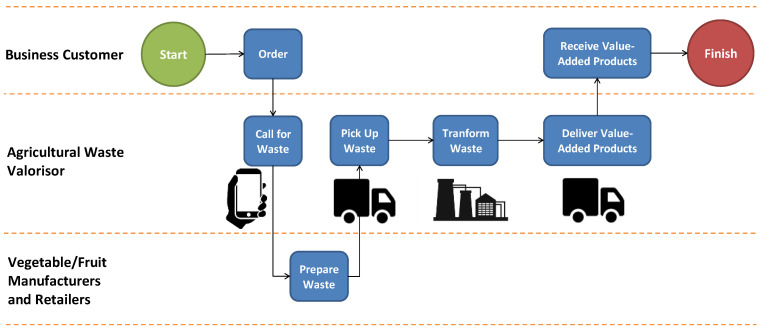
Swimlane diagram of digital waste management platform. Source: Figure created by authors 2023.

Therefore, developing a digital platform can help solve inefficient agri-waste management by collecting and managing waste data and providing possible business partners that could use agricultural waste as a high-value-added product in the future. The swimlane diagram of this digital platform is demonstrated in Figure 1. This digital platform helps to gather the network of vegetable and fruit retailers in fresh markets and processed food manufacturers that have food wastes such as peels, seeds, and flesh. It allows businesses that need materials transformed from these wastes (such as paper, bioplastic, vegan leather, plywood, and straws, among others) to order their value-added materials/products from this platform. The biomaterial pilot plant acts in the middle and is responsible for transforming these agricultural wastes into value-added materials/products. This platform also helps to manage the monetary transaction from the beginning, including collecting money from the business customers, distributing money to waste sellers, managing logistics, and the final stage, which is to send the materials/products to the business customers.

However, the idea of a circular economy is still relatively novel in Thailand, particularly among food merchants and farmers. It is necessary to have a profound understanding of the individuals that would use this technology. Based on previous research, digital platform developers can better comprehend user behaviors when categorizing these users into specific segments and analyzing the factors affecting their use intention [9,16]. However, only a small number of studies have focused on the factors influencing these behavioral intentions and the segmentation of digital platform users for a circular economy. In addition, this study contributes to the current body of the literature by analyzing the moderating effect of demographic segments on a number of factors that influence behavioral intentions. On the basis of their gender, age, and annual income, the users were divided into two distinct demographic subgroups. The research framework was based on the Unified Theory of the Adoption and Utilization of Technology (UTAUT2), which posits the determinants of technology adoption, including performance expectancy, effort expectancy, social influence, facilitating condition, hedonic motivation, price value, and habit [17]. The UTAUT2 was justifiable in this context because evidence revealed that the UTAUT2 has been employed in research on food and agricultural technology to predict users’ behavioral intentions [18,19,20]. An additional academic contribution is that the UTAUT2 can be expanded with more variables, such as trust and privacy [21], to enrich the research framework and provide novelty for technology adoption concerning circular-economy-based digital technology.

With this novel platform, this study contributes to bridging the gap of factors that influence users’ behavioral intentions toward a digital waste management platform. Explicitly, this study aimed to deeply understand the digital adoption behaviors of users who are vegetable and fruit retailers and processed food retailers. First, we classified the users using the multivariate demographic segmentation approach. Then, we employed the multigroup structural equation modeling approach to explore the factors influencing their behavioral intentions towards adopting digital technology based on the UTAUT2 model.

## 2. Literature Review and Hypothesis Development

The structural equation model (SEM) has been utilized in many circular economy studies to show the link between customer/user perceptions, attitudes, and behavioral intentions [22,23,24,25,26]. These papers have discussed firm structures, mechanisms, and performances. However, a digital circular model has rarely been discussed. In the present study, a review of the relevant literature was carried out to pinpoint the relevant factors, models, and determinants. In order to achieve the objectives of this investigation, the UTAUT2 model was utilized using the SEM framework.

### 2.1. The Unified Theory of Acceptance and Use of Technology 2: UTAUT2

Recent research has utilized the UTAUT2 to explain users’ behavioral intentions towards adopting digital platforms in the food and agricultural industry [18,19,20]. The UTAUT2 is extended from the Unified Theory of Acceptance and Use of Technology (UTAUT), which is based on sociology and psychology and is one of the most recent theories of how people accept new technologies. It is a development of prior technology acceptance models, which have been widely used in studies on how people embrace new information technologies. The UTAUT is based on eight models of technology acceptance [17] that have been used in a number of similar studies on consumer technology uptake [27,28,29,30]. Referring to a previous study, the UTAUT has been used often in the social sciences [31]. The UTAUT is a theory that can be applied broadly and contains model components that are accurate and reliable. These model elements are used for projecting and explaining user acceptance behaviors [32,33]. It has been demonstrated that the theory is capable of explaining a significant amount of variability in a wide range of technological adoption and usage behaviors [17,34,35]. The UTAUT explains that technology acceptance can be determined by performance expectancy, effort expectancy, social influence, and facilitating conditions, while demographic variables such as gender, age, and income moderate this relationship [17].

The UTAUT2 added three more determinants of technology acceptance to the original UTAUT model, including hedonic motivation, price value, and habit [36]. The UTAUT2 was utilized in this research because it comprises acceptance and use models (adoption) of cutting-edge information technology that have been tested and verified in previous research. As a result, in the quest for an acceptable model that embraces practically all the components driving users’ behavioral intentions towards a fruits and vegetables waste management platform, the UTAUT2 was the most acceptable theoretical foundation to supply the conceptual model utilized in this research. The main constructs in the UTAUT2 are: performance expectancy (PE), effort expectancy (EE), social influence (SI), facilitating condition (FC), hedonic motivation (HM), price value (PV), and habit (HB), plus two extended variables: trust (TR) and privacy (PR), which were proposed as direct determinants of the behavior intentions of users. In addition, gender, age, and income were assumed to influence this usage intention and behavior.

Due to the high amount of garbage generated by fruit and vegetable waste from sellers and farmers, a prototype of the platform concept was created to serve companies for purchasing their fruit and vegetable waste. The platform would collect information from the merchants and farmers in each area who desired to sell their garbage. The platform would then convert this vegetable and fruit waste into usable items or transform them into biological products from plant waste, such as paper and organic leather. However, there have been few studies in Thailand on platforms that act as an intermediary for acquiring leftover vegetables and fruits, as well as the transformation process of these leftovers into high-value products. Consequently, the researchers aimed to investigate the elements that impact personal preferences for utilizing this platform and acting as an intermediary in the trade of vegetable and fruit waste from merchants and farmers.

### 2.2. Performance Expectancy (PE)

The possibility that this new system and application would assist users in meeting their requirements and goals in a more practical and efficient manner is referred to as the performance expectancy [17]. In conformity with previous studies, an important relationship between performance expectancy and behavioral intention was revealed [37]. If customers feel that a new system would save them more time and effort than the existing systems, they are more likely to be satisfied and plan to utilize the new system [17,38,39,40]. Regarding food-related performance expectations, the inherent mobility and adaptability of mobile food-ordering applications have facilitated more convenient interactions between clients and restaurants. For instance, when using a mobile food-ordering app, a customer may visit any restaurant at any time, have a broad choice of food selections, obtain sufficient information, and make orders without traveling. Given issues such as traffic or wasting time looking for parking, mobile food-ordering applications are extremely significant. Therefore, if a customer experiences a high degree of utilitarian value in using such creative applications, they are more likely to be content and delighted with their experience. This is because of the increased likelihood that they will use these apps again. According to the research of Yeo et al. (2017), customers’ propensities to utilize online food-ordering systems are significantly influenced by the usability of the system, which is a factor that is analogous to the performance expectancy [41].

**Hypothesis** **H1.** *Performance expectancy will positively influence users’ intentions to use the fruit and vegetable waste management platform*.

### 2.3. Effort Expectancy (EE)

Effort expectancy evaluates the degree of usability associated with the usage of information technology. Venkatesh et al. (2003) defined effort expectancy as the degree of simplicity involved in using an information system [17]. It indicates the extent of user expectations that the usage of such platforms would not need physical and mental exertion. Existing research has shown that effort anticipation favorably modifies behavioral intentions [34,42,43]. Performance expectancy also has a role in mediating this effect [35]. This implies that people will have high expectations for the outcomes that are expected of them and be more open to using technology if they believe it is simple to use. However, the level of effort that may be anticipated is proportional to the extent to which users sell their waste products via the platform. This is because the ease with which customers may obtain essential information by using a trading platform in the shortest amount of time possible has a high probability of influencing the amount of time these customers spend using the platform. Because of this, clients who learn how easy it is to use the platform for trading may not be reluctant to use it.

With this proposition that relates to effort expectancy, its considerable influence on consumer desires to adopt mobile food-ordering applications has been experimentally shown [44]. The amount of time and effort required from customers may also reflect how simple or difficult it is to use these programs for ordering food via mobile devices. For this reason, it can be said that consumers will be content with their experience of utilizing these applications as long as they judge the effort and complexity to be low. According to the findings of Amin et al. (2014), there is a significant correlation between the simplicity of using a mobile app, the amount of effort needed, and the level of customer satisfaction [45].

**Hypothesis** **H2.** *Effort expectancy will positively influence users’ intentions to use the fruit and vegetable waste management platform*.

### 2.4. Social Influence (SI)

Social influence is a marketing term that refers to the ability to influence the thoughts of people in a social network online. The greater a person’s influence, the more enticing they are to companies and other individuals attempting to promote or sell an idea or product. A second definition of social influence was provided by Turner and Oakes (1986) [46], who referred to it as “the processes by which individuals directly or indirectly affect the attitudes, feelings, and behaviors of others”. Information about other people is a prerequisite for exerting social influence, and direct contact with these individuals is not always necessary for this to take place [47]. In the past, a person’s social influence was restricted to the individuals in their immediate social circle; however, the internet and other social media platforms have significantly increased the scope of a person’s social impact [48]. It is the conviction held by a person that other people should take advantage of their available technological resources [17]. In the early stages of a person’s interaction with new technologies, social influence is particularly significant, because the information received from the people around them contributes to the usefulness of a specific technology [21]. These experiences give a more realistic foundation for an individual’s continued use of technology, despite the fact that its use reduces over time and becomes outmoded as technology advances.

Researchers have stated that consumers’ food waste habits are complicated as a result of the interplay between numerous home activities, and that influencing these behaviors is crucial [49,50]. The social impact theory might be one way that people learn from one another, resulting in attitude and behavior modification [51]. Households may be able to lessen their effects on the natural environment if they are able to harness this for pro-environmental behavior changes.

**Hypothesis** **H3.** *Social Influence will positively influence users’ intentions to use the fruit and vegetable waste management platform*.

### 2.5. Facilitating Condition (FC)

The phrase “the extent to which an individual feels that an organizational and technical infrastructure exists to support the operation of the system” is what is meant when one refers to “facilitating conditions”. In e-commerce, the term “facilitating condition” refers to resources such as a computer, tablet, mobile phone, internet access, and the ability to make payments online, as well as the expertise needed to install and use essential software [17]. Earlier studies on the desirability of platforms have shown that users’ perceptions of their convenience (i.e., having the tools and help necessary to use technology) have a direct influence on their behavioral intentions to use the technology [52]. Consumers are motivated to use mobile shopping technologies [53], online banking [54], online airline ticketing [55], and also mobile payment services when they have the technology and infrastructure support they need [56]. This shows that facilitating conditions influence the behavioral intentions of users in a positive manner. Additionally, consumers with adequate financial resources are more at ease and satisfied when utilizing technology. Users that are more proficient with a platform and those that have a higher technical expertise are also likely to continue using it.

**Hypothesis** **H4.** *Facilitating conditions will positively influence users’ intentions to use the fruit and vegetable waste management platform*.

### 2.6. Hedonic Motivation (HM)

The level of enjoyment one derives from using cutting-edge technology is known as hedonic motivation, and it has been acknowledged as being crucial for the adoption and application of technology [17]. This idea has been defined as perceived enjoyment in various theories or models of technological acceptance [36]. If technology is delightful and entertaining to use, its users can derive enjoyment from it [28]. According to Leong et al.’s [57] study on mobile entertainment, consumers are eager to use mobile entertainment if it will bring them happiness and joy. Based on previous research, customers will adopt new technology when they believe that using it would make them more comfortable, happy, and amused. People will be more inclined to use a platform if they think utilizing it will be pleasurable [40]. As a result, hedonic motivation is defined as the moment when individuals want to use a platform consistently, and their continued behavior is based on knowledge or experience [17]. Moreover, it has been established that hedonic motivation is a crucial component of the behavioral intention to adopt mobile internet in a consumer setting [36]. Diverse genres of mobile applications, such as games, can provide users with enjoyment and diversion. If a customer believes that using mobile applications is fun, he or she will be more inclined to utilize them. In recent years, people have become more reliant on food delivery as a result of the present pace of life and the possibility of finding additional eateries provided by this food delivery [58].

**Hypothesis** **H5.** *Hedonic motivation will positively influence users’ intentions to use the fruit and vegetable waste management platform*.

### 2.7. Price Value (PV)

Compared to organizational settings, the use of technology in the context of the customer may incur additional financial costs. Consequently, customers can cognitively weigh the advantages of adopting new technologies against the costs associated with their use. Customers will be more eager to adopt these new technologies if their price-to-value ratio is more favorable. This, in turn, requires that customers perceive using this technology as more useful and valuable than the monetary expenditure that is spent as a result of using the technology [36]. Based on the findings of earlier studies, the economic value of a product is a crucial component in the formulation of behavioral intentions when deciding on customer acceptance and the usage of technological products [40]. When utilizing a program, users will continue to weigh its associated costs. If the advantages outweigh these costs, the user will continue to use the program over time. This is consistent with the findings of Hew et al. [59], who said that price value influences behavioral intentions [59]. Among the elements responsible for processing food delivery apps, demand, price changes, discount offers, the availability of pricing comparisons, and the simplicity of selection are the most prominent [60,61]. Because of the burden that it places on their finances, large families normally make fewer purchases of home-delivered meals than families of a smaller size do; large families are more likely to take advantage of various discounts and deals [62]. Similarly, Koiri et al. (2019) suggested that restaurants should provide customer incentives such as discounts or cash-back offers as a way of fostering a good attitude and take into account that people do not want to pay online [63]. Pricing value has a positive impact on the willingness to use technology when it is thought that the advantages of using this technology surpass its monetary expenditures [36].

**Hypothesis** **H6.** *Price value will positively influence users’ intentions to use the fruit and vegetable waste management platform*.

### 2.8. Habit (H)

Habit refers to the belief that behavior is automatic in an individual [64]. Previous studies have discovered that habit has both direct and indirect effects on behavioral intentions [36]. Food habits are established over time and grocery shopping could quickly become an established part of one’s weekly schedule [65]. Most food waste studies incorporate consumers’ purchasing habits, which may be a key driver in identifying their food waste behaviors [66,67,68]. According to Stefan et al. [69], shopping behaviors that lead to overbuying or the purchase of unexpected products significantly affect how individuals dispose of their food. Most people’s shopping regimen includes buying more than they need [70]. Buying in excess and getting carried away with exceptional promotions such as buy-one-get-one-free or mixing multiple goods can lead to food waste [71,72]. Furthermore, multiple studies have demonstrated that people who prepare shopping lists and buy just what they need waste less food [73,74].

**Hypothesis** **H7.** *Habit will positively influence users’ intentions to use the fruit and vegetable waste management platform*.

### 2.9. Trust (TR)

Zhou et al. (2010) defined trust as “the degree of ease associated with the use of the system” [35]. Trust is an enabler in distributed systems. It enables internet commerce and safe agent-based apps. Despite the need to standardize trust and its related concepts, many researchers have employed a very precise meaning of trust as being connected to authentication or the capacity to pay for purchases. Prior studies have shown that trust does not have much of an effect on a person’s intentions toward their actions [37]. Trust is a crucial consumer factor in other food situations. However, one recent study investigated the reasons that trust functions as an obstacle to platform adoption, which revealed that quality and interface concerns are important challenges that customers may experience while using meal delivery services [75]. Thereby, barriers to utilizing meal delivery apps might be related to the technological characteristics of an app, as well as the actual experience in terms of the quality of the food and the service that is provided. Applications specializing in food delivery, in particular, are examples of technology-based services that enable users to order food for consumption physically. These apps may present users with challenges numerous times, both before and after the transaction is completed. This is consistent with prior studies that have shown that mobile app services with online-to-offline settings, such as food delivery applications, app functionality, and offline usage, are critical [76].

**Hypothesis** **H8.** *Trust will positively influence users’ intentions to use the fruit and vegetable waste management platform*.

### 2.10. Privacy (PR)

The successful adoption and adaptation of technology relies heavily on the protection of personal information. This is the extent to which an individual’s right to control the information collected on them by third parties is protected [77]. Previous research has clarified that a user’s inclination to utilize an application is largely determined by their level of trust [78]. Previous studies have discovered that privacy has a significant impact on behavioral intentions [79]. In this day and age of the internet, the protection of private information is of the utmost importance, as it poses a risk to the integrity of online financial transactions, as well as the preservation of personally identifiable information [80]. When it comes to the usage of mobile apps for a variety of tasks, such as online shopping or online food delivery [81], particularly financial operations [82], users place a high value on the protection of their private information as a crucial consideration. If the safety of the transaction can be guaranteed, users of apps for online food delivery will have a greater propensity to utilize such services [77,82].

**Hypothesis** **H9.** *Privacy will positively influence users’ intentions to use the fruit and vegetable waste management platform*.

### 2.11. Behavioral Intention (BI)

The term “behavioral intention” is used to describe the degree to which a person has made up their mind about whether they will participate in a certain future behavior [83]. Perceived usefulness and trust may play important roles in establishing the behavioral intent to adopt mobile commerce [84]. Researchers have proposed that behavioral intentions to utilize online platforms for home fights is highly affected by both performance and effort expectations. Furthermore, social influence has a significant impact on behavioral intentions towards embracing mobile use [85]. Previous research has found that several independent factors can impact these behavioral intentions. As a result, the dependent variables chosen would be determined by the theory employed and the study situation. Behavioral intentions, as defined by Ajzen (2015), are what show how much effort people are willing to put into accomplishing a job [86]. In contrast, Malhotra and McCort (2001) said that marketing scholars still place a premium on learning more about consumers’ future plans [87]. Despite this, a customer’s purpose continues to stand out as a distinctive feature that plays an extra substantial influence on consumer behaviors. To better predict a customer’s actual behavior on the path leading up to a specific action, it may be useful to first obtain some insight into that customer’s reasons [88].

A person’s conceptual framework and level of readiness to act, in relation to food waste, are both internal and contextual factors. Behavioral intention is a significant predictor of actual behavior [86]. As a result, the hypothesis accounts for the positive impact of meaning on food waste management actions [89]. In Shenzhen, China, 491 construction workers were surveyed regarding their thoughts and behaviors about construction waste. Waste reduction behavior is affected by individuals’ intentions to do so, as shown by recent research [90]. Customers’ food waste habits can be better understood, and evidence of the positive effect of behavioral intentions on actual waste reduction can be provided [91].

### 2.12. Moderation Effects of Demographic Variables

The UTAUT2 was selected as the main theory in this research. Hence, demographic variables such as age, gender, and income would moderate the relationship among the main UTAUT2 variables. This section reviews the roles of age, gender, and income, which have an impact on the use of technology.

Inglehart (2015) first presented the Generational Cohort Theory as a method for segmenting a population by generation [92]. Consequently, generational cohorts tend to share similar beliefs, values, and attitudes because they share the same early adulthood experiences and social, political, and economic events (ages 17–24). These experiences and occurrences affect their core beliefs in areas such as tolerance, money, jobs, and sexual conduct [93].

As was mentioned earlier, the participants in this study came from four different generations: baby boomers, who were born between 1946 and 1965; Gen X, who were born between 1966 and 1980; Gen Y, who were born between 1981 and 1995; and Gen Z, who were born between 1996–2013 [94,95].

Presently, most of the population comprises older individuals, who are classed as baby boomers. During the formation year in terms of social, political, and economic issues, baby boomers are generally prosperous. Because of these experiences, the baby boomer is ideal. They are positive, self-confident, and communicate well. They value education and are avid product users [96]. The baby boomer generation is frequently described as the least tech-savvy of the generations when studying information technology and media [97]. Although their rate of mobile data and technology use continues to rise [98,99], they continue to lag behind younger generations. Baby boomers tend to have high intentions towards purchase upcycled foods compared to Gen X. Due to lifestyle differences, baby boomers seem to support upcycled food [100].

Unlike younger generations, Gen X is not surrounded by and preoccupied with technology; therefore, they are considered “digital natives”. They must invest a great deal of time and energy into acquiring digital abilities [101]. Members of Gen X are seen as being gloomy, cynical, socially insecure, and lacking in established conventions, which is in stark contrast to the optimistic attitude of the baby boomer generation [102]. A previous study found that Gen X have the lowest intentions towards purchasing upcycled foods, due to a decrease in the perceived quality of these upcycled foods [100].

The expansion of the economy occurred along with the maturation of members from Generation Y. The rapid development of new technologies (such as the introduction of the internet and other forms of social media), globalization, and popular culture impact people’s way of life [103]. In contrast to its predecessor, Generation X, the generation known as Generation Y is seen as being upbeat and self-assured. They respond by taking the necessary steps to fix a problem when it occurs. Because of their superior strength and speed, members of Generation Y are capable of performing many tasks at once [104,105]. In terms of social connections, interests, friendships, and civic activities, technology plays an integral role in their lives [106]. Because its members are digital natives whose lives revolved around technological advancement, Gen Y is commonly referred to as “the Internet Generation”. According to previous research, members of Gen Y consider buying organic foods because of concerns about their health, and their trust in organic food impacts their purchasing intentiosn. However, higher prices reduce Gen Y’s organic product purchasing intentions [100].

Gen Z is a generation that has a significant impact on the nation’s economy and society. Even though Thailand has entered an aging culture, Generation Z advise the elderly in their product purchases. The Hakuhodo Institute of Life and Living ASEAN: HILL ASEAN, a major advertising agency from Japan, conducted a study of such consumer behaviors in six ASEAN countries and found that members of Gen Z, most of whom have Gen X parents, are people born in the social media and internet era. They receive a lot of information from the internet. They objectively consider the words and actions of previous generations. From a broad perspective, they want to solve the social problems that may arise from previous generations by giving equal importance to themselves, their families, and those around them, being ready to work together to solve these problems, even if there are differences between them. As a result, previous research has shown that Gen Z’s dietary preferences can be influenced by a good impression of diet and food [100].

Gender has been found to impact behavioral intentions towards using technology. Gender is a social framework that differentiates between the limitations and possibilities based on sex categories, and this differentiation has ramifications on various levels [107]. Gender is built in several different ways, including through differences in women’s and men’s access to protective factors (such as resources) and differences in their exposure to risk factors (such as stresses). Men and women are satisfied with different things. For instance, a recent study found that gender moderates the relationship between performance expectancy, facilitating conditions, social influence, and behavioral intentions towards accepting e-training technology [108].

Additionally, income may play a moderating role in the technology acceptance model [109]. This study examined customer intentions towards adopting an e-ticket airfare platform. This study found that the perceived usefulness of the older group with middle-to-high incomes was lower than that of the younger group with low incomes. Unsurprisingly, younger respondents reported a greater perception of perceived ease of use than older respondents. The younger respondents were more sensitive to price than their older counterparts. Young people had a greater perception of hedonic motivation than older individuals. Lastly, younger respondents had greater behavioral intentions towards purchasing airfares through e-commerce than older respondents.

Therefore, we hypothesize that, together, demographic variables (age, gender, and income) have a moderating effect on this UTAUT2 model. This moderating effect is presented using a multivariate demographic cluster analysis, signifying the academic novelty in this study. We also decided to omit the use behavior construct from this model because this platform is a concept prototype, so no actual use can be investigated. Additionally, the constructs of trust and privacy were added to the model, indicating an additional theoretical contribution to this study.

**Figure 2:**
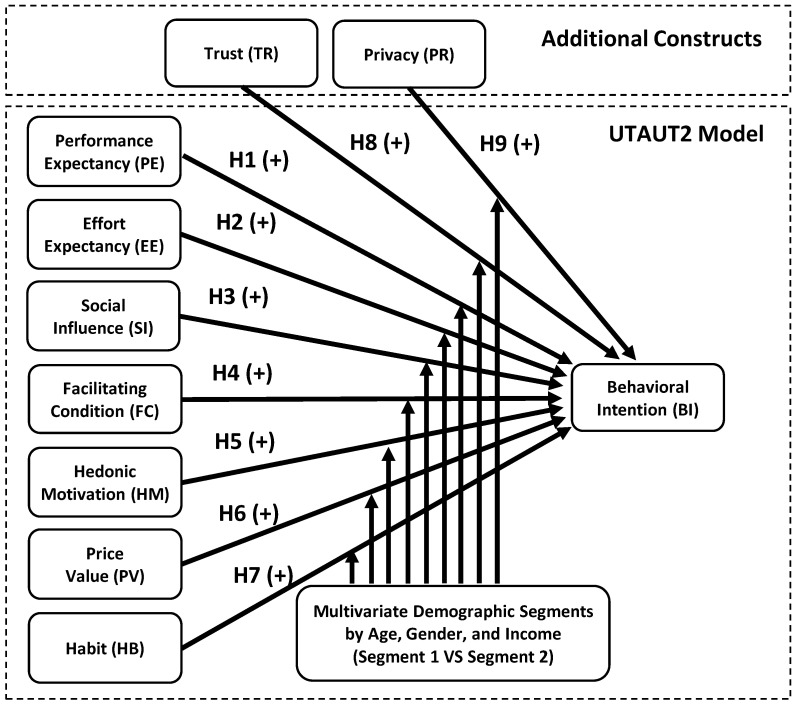
Research framework. Source: Figure adapted by authors (2023).

Taking into account the literature studies and theories, Figure 2 also depicts the conceptual model of this investigation.

## 3. Methodology

The method in this study mainly employed a quantitative analysis centered around the UTAUT2 theory. This section involves the sampling, data collection, and data analysis.

### 3.1. Sampling and Data Collection

For the sample size in a data analysis, it is suggested that no particular criterion be adhered to when selecting a specific sample size for a confirmatory factor analysis (CFA), the first stage of structural equation modeling (SEM) [110]. Tabachnick and Fidell (2007) [111] speculated that CFAs depend on sample size and might be less stable when examined using a small sample. If a structural model contains fewer than seven constructs, a minimum sample size of 300 is advised [110]. On the contrary, Kline (2016) suggested 200 observations as the standard sample size for an SEM investigation [112]. As a result, the researchers intended to collect data using a structured questionnaire obtained through intercept surveys with 350 respondents.

This study utilized a combination of quota and purposive sampling techniques. Obtaining data from a group that is representative of the whole is the purpose of this sampling technique [113]. The research takes into account the proportions of the quota responders drawn from the whole population of farmers and sellers in the northeast of Thailand (350 respondents: 200 farmers and 150 sellers). It was decided to employ the quota sampling approach in order to select an equal number of fruit and vegetable farmers and sellers from the provinces with sizable agricultural areas and fresh markets, including Nakhon Ratchasima (*n* = 70), Khon Kaen (*n* = 70), Ubon Ratchathani (*n* = 70), Sri Saket (*n* = 70), and Roi-et (*n* = 70) [114]. This purposive sampling method was employed because we focused only on the respondents who were vegetable or fruit sellers and farmers from the selected markets and farms in those provinces.

An intercept survey was used as the method for data collection because it was the most effective for gathering perception data from the respondents located in the designated areas. This method enabled respondents to complete the questionnaire in a single setting. Consequently, the quality of their feedback was enhanced by reducing distractions [115]. The respondents, who were farmers and merchants, were purposively asked to respond to this questionnaire. Furthermore, in order to avoid ambiguity that might contribute to response bias, the researchers only allowed the respondents to reply to the questionnaire once. First, the surveyor began by asking the respondents whether they were farmers (or retailers) who were potential users of this digital platform to ascertain the survey. The questionnaires used were confidential and provided the respondents with anonymity. Before responding to the questions, the respondents were informed of the research objectives. All the respondents had to be at least 20 years old, able to comprehend Thai, and willing to answer the questions. They were asked about their age, Thai reading ability, and participation intentions. This questionnaire could take about 5–10 min to complete. If they felt uncomfortable responding to the questionnaire, the respondents were free to decline at any time. This survey was conducted following the approval of the Khon Kaen University Ethics Committee for Human Research (HE643227).

Despite this, we were able to utilize data from only 326 respondents (200 farmers and 126 sellers) from the previously determined locations in northeastern Thailand. Out of 100 percent of the questionnaires distributed, the usable rate was only 93.14 percent. This was because some respondents did not complete all the questions, while some took the questionnaires but never returned them. The data curation and utilization were governed by human research ethics considerations to ensure the confidentiality of the respondents’ information. Before responding to the questionnaires, the respondents were instructed about using this digital platform. The process flow of this platform was also described according to Figure 1.

The questionnaire was divided into two main parts (see Appendix A): demographic profiles and user perceptions. In the demographic part, there were three questions that asked about age (the Gen Z ages were between 20–27, Gen Y ages were between 28–41, Gen X ages were between 42–56, and baby boomer ages were between 57–76 [94,95]), gender (male and female), and income (less than BHT 15,000, BHT 15,000–20,000, BHT 25,001–30,000, and more than BHT 30,000). For the perception part, the questions were adapted from Venkatesh et al. [36] and Alam et al. [21]. The questionnaire referenced the determining elements and models. To quantify the constructs, this study made use of a five-point Likert scale, with 1 representing strong disagreement and 5 representing strong agreement.

### 3.2. Data Analysis

In this study, we first addressed the issue of common method variance (CMV) before analyzing the data using the statistical tools. CMV occurs when variables within the same model are studied using the same method or same source, leading to systematic error variations and biasing the evaluated relationship. In order to investigate CMV, this study used Harman’s single-factor test, originally developed by Podsakoff et al. (2011) [116]. This allowed the researchers to collect both dependent and independent variables from the respondents. The results showed that the total variance was 39.325%, which was less than the 50% criterion [116].

To categorize the respondents by age, gender, and income, the researcher employed a multivariate cluster analysis. To begin, we used a hierarchical cluster analysis to build the clusters by increasing the levels of similarity and dissimilarity among the groups [117]. Secondly, the hierarchical cluster result was validated using a centroid cluster analysis. Lastly, the squared Euclidean distance was employed to minimize the average squared distance between the values that were observed, and the value was estimated [118]. By observing the dendrogram, the Elbow method was used to determine a suitable number of clusters. As a follow-up, a series of chi-square tests were run to confirm any significant variations identified among the clusters in terms of the segmentation by demographics. Next, we conducted a *t*-test to compare the mean perception scores of Segment 1 and Segment 2 and to check whether there were any differences between the clusters [119,120]. To ascertain the normality of the data set, we used the Kolmogorov–Smirnov, Shapiro–Wilk, Skewness, and Kurtosis tests to ensure that the data were normally distributed. Both the Kolmogorov–Smirnov and Shapiro–Wilk tests yielded statistically significant results (*p*-values < 0.001). Furthermore, the perception scores for the Skewness and Kurtosis values fell within the acceptable range of 1.00 to 1.00. Therefore, the normality conditions were met by this data set.

The structural equation modeling (SEM) approach was used to analyze this study’s data, utilizing the statistic program IBM SPSS Amos 26. Among the statistical techniques in SEM are confirmatory factor analysis (CFA), path analysis, causal modeling using latent variables, analysis of variance, and multiple linear regression [121]. This method was utilized to explore user behavioral intentions in food and agricultural research [9,16,18,19,122] In practice, two steps of SEM were used to evaluate the model estimate [123]. First, the outer CFA model was assessed to establish the validity and dependability of the relationship between each indicator and its variable. The goodness of fit (GOF), discriminant validity, and convergent validity were all examined at this stage. The second stage consistsed of a GOF study of the inner structural model to determine the overall structural validity. The final phase of this technique involved the use of a multigroup moderation analysis to evaluate the moderating impact of the multivariate demographic segmentation on the structural relationship revealed by the UTAUT2 hypothesis. Using a measurement invariance (MI) analysis with the demographic segment as a moderating factor, the sample was divided into two groups in this stage.

## 4. Results of the Study

### 4.1. Multivariate Demographic Segmentation

According to the cluster analysis results from Table 1, we divided the users into Segment 1 (*n* = 122) and Segment 2 (*n* = 204). The chi-square test demonstrated that gender was an insignificant variable for the demographic segmentation (*p*-value = 0.163 > 0.01). Most respondents in Segment 1 were people from Generation X and baby boomers. Furthermore, most people in this group had low incomes and were fruit and vegetable retailers, but the higher-income earners were manufacturing officials. However, in Segment 2, Gen Z and Gen Y members made up the bulk of the participants, who clearly earned less than BHT 15,000. This result concluded that the users were classified into two different groups: (1) older people with various income ranges, and (2) young people with low incomes, where gender was ineffective in classifying these segments. As for Segment 1, the respondents’ monthly incomes were less than BHT 15,000.

Next, the perception scores of Segment 1 (older people with various income ranges) and Segment 2 (young people with low incomes) were compared. Table 2 shows that, compared to Segment 1, the users in Segment 2 had higher mean scores across the board with regard to performance expectancy, effort expectancy, social influence, habit, and behavioral intentions. In contrast, the Segment 1 respondents averaged a greater pricing value and a higher level of privacy than the Segment 2 respondents. However, regarding facilitating conditions and hedonic motivation, comparing the mean scores of Segments 1 and 2 was inconclusive.

After applying *t*-tests across the two groups, there was a statistically significant difference between the groups’ mean scores, given *p*-values of <0.05, <0.01, and <0.001. Table 2 demonstrates that Segment 2 tended to have a greater perception that the platform may increase productivity (PE3) than Segment 1. Segment 2 also expected the platform to be easier to learn than Segment 1 (EE1). Moreover, the perceptions of social influence regarding referrals (SI1), influencers (SI2), and respected people (SI3) in Segment 2 seemed to be better off than those in Segment 1. The perception of habits, such as addiction (HB2) and routine (HB3), in Segment 2 tended to be higher than those in Segment 1. Segment 2 also expected to have the necessary knowledge to utilize the platform more than Segment 1 (FC2). In contrast, the *t*-test results show that Segment 1 expected to have more support resources to help them utilize the platform (FC1) and required more privacy (PR4) than Segment 2.

There are two primary steps in conducting a statistical test with SEM: the measurement model (CFA) and the structural model [123].

### 4.2. Confirmatory Factor Analysis (CFA)

A CFA was utilized in order to conduct tests on the measurement model. Within the scope of this investigation, the model was evaluated with regard to its internal consistency, convergent validity, and discriminant validity. The CFA was carried out by coupling all the constructs to their respective covariances. Before testing could begin, every construct had to have its own set of manifest variables. The goodness of fit (GOF) of the entire connection might be developed based on the covariances among the errors that occurred within the same constructs. This analysis can be elaborated as follows.

#### 4.2.1. The Goodness of Fit (GOF)

In Table 3, we see the GOF measurements and thresholds. All the metrics met or exceeded the established standards; hence, the outcomes were acceptable. The values for the comparative fit index (CFI; 0.913), incremental fit index (IFI; 0.0914), TLI Tucker–Lewis index (TLI; 0.900), and root mean square error of approximation (RMSEA; 0.071) all exceeded their predetermined thresholds for goodness of fit. In terms of the GOF condition, the stated thresholds were a CMIN/df less than 3.00, CFI larger than 0.900, IFI greater than 0.900, TLI greater than 0.900, and RMSEA less than 0.100.

#### 4.2.2. Convergent Validity

A comparison of the model’s outcomes with the predetermined thresholds for the fit index was used to analyze the convergent validity. Cronbach’s alphas, average variance extracted (AVE), and composite reliability (CR) were utilized to analyze the measures’ degrees of consistency against the recommended thresholds for an AVE and CR of 0.05 and 0.70, respectively. Accordingly, the thresholds for the convergent validity measurements and the derived indicators are listed as follows.

Table 4 shows that, when the calculated measures were compared to the thresholds, the values for the performance expectancy, effort expectancy, social influence, facilitating condition, hedonic motivation, price value, habit, trust, privacy, and behavioral intention all passed the convergent validity criteria. In terms of the constructs, each and every indicator reached a level of statistical significance of <0.001, and all the AVEs were greater than the thresholds (AVE > 0.50). On the other hand, the Cronbach’s alphas and CR values were all higher than 0.7, which indicated that all the indicators included in this measurement model met the criteria for the convergent validity.

#### 4.2.3. Discriminant Validity

The discriminant validity of a test or measurement is defined as the degree to which it varies from another test or measurement with the same underlying idea. To evaluate this component, the Fornell and Larcker criteria were utilized to compare the square root AVEs (located on the diagonal) and the correlations of the various matrices. Table 5 shows that the square root of each bolded AVE was bigger than the off-diagonal correlation coefficients. This implies that all of the variable compositions may theoretically measure the unique constructs, and the outcome of this was satisfactory. Furthermore, each AVE’s square root was greater than the off-diagonal correlation coefficients.

In addition, Henseler et al. (2015) used the heterotrait–monotrait (HTMT) ratio technique to examine discriminant validity [124]. This was performed because the Fornell and Larcker (1981) criterion was criticized for its lack of reliability in addressing the distinctiveness between latent variables [124]. Specifically, this problem was addressed by Henseler et al. (2015). HTMT values greater than 0.90 suggest the presence of discriminant validity between the associated latent variables. According to Table 5, each value on the HTMT is lower than 0.90, indicating that it satisfies the criteria for the discriminant validity.

### 4.3. Structural Model

We come to demonstrate the SEM analysis after achieving the prerequisite for the reliability and validity. According to Table 6, as Hu and Bentler (2009) suggested, the goodness of fit criteria mostly supported this structural model [125]. According to Table 7, the structural model’s test results supported H3, H4, and H7 to H9 at a significant level of 0.05 or less. This showed that the correlations between the constructs were statistically significant. The analysis was based on the following concepts: performance expectancy (PE), effort expectancy (EE), social influence (SI), facilitating condition (FC), hedonic motivation (HM), price value (PV), habit (H), trust (TR), privacy (PR), and behavioral intention (BI).

The results rejected H1, which hypothesized that performance expectancy would positively influence the users’ intentions to use the fruit and vegetable waste management platform. This result demonstrated an explicitly contradictory result with a standardized loading of 0.054 (*p*-value = 0.403).

H2 was rejected, which meant that effort expectancy had no effect on the users’ intentions to use the fruit and vegetable waste management platform. With a standardized loading of −0.012 (*p*-value = 0.857), this result showed a conflicting outcome.

H3 was supported, which recommended that social influence would positively influence the users’ intentions to use the fruit and vegetable waste management platform, with a standardized loading of 0.222 (*p*-value < 0.001). This study demonstrated that the inclination to use the platform was affected by social factors such as close friends, family, friends, and notable persons.

H4 was supported, which recommended that, with a standardized loading of 0.143 (*p*-value = 0.030 < 0.05), facilitating conditions would positively influence users’ intentions to use the fruit and vegetable waste management platform. This result was consistent with the hypothesis that users’ perceptions of convenience have a direct influence on their behavioral intentions to use technology. This indicates that consumers are motivated to use mobile shopping technologies when they have the necessary support from both the infrastructure and the technical side.

Moreover, the outcomes rejected H5, which stated that hedonic motivation does not influence the users’ intentions to use the fruit and vegetable waste management platform, with a standardized loading of 0.085 (*p*-value = 0.227).

The outcomes also rejected H6, which asserted that price value would positively influence the users’ intentions to use the fruit and vegetable waste management platform. With a standardization factor of 0.119 (*p*-value = 0.126), this result was contradictory.

H7 was supported, indicating that habit would positively influence the users’ intentions to use the fruit and vegetable waste management platform, with a standardized loading of 0.259 (*p*-value < 0.001). This study’s findings recommended that the routine activities of users had an effect on their intentions to use the platform.

H8 was supported, which hypothesized that trust would positively influence the users’ intentions to use the fruit and vegetable waste management platform, given a standardized loading of 0.158 (*p*-value = 0.046 < 0.05). The results of this survey showed that the users of the platform were quite conscious of the trust that is provided by the platform.

H9 was supported, which recommended that privacy would positively influence the users’ intentions to use the fruit and vegetable waste management platform, with a standardized loading of 0.365 (*p*-value < 0.001). This study demonstrated that the platform users had a high level of awareness regarding the safety of their personal data.

### 4.4. Multigroup Moderation Analysis

Measurement invariance (MI) is a method that utilizes indicators to determine a latent characteristic across all the respondents in Segments 1 and 2 [121]. The MI technique has three parts: configural invariance, metric invariance, and scalar invariance. The distinction between full and partial MI is based on the findings from the following investigation. When only the conditions for configural invariance and metric invariance are met, partial invariance can be considered to exist. Despite this, full measurement invariance is established after both partial MI and scalar invariance are taken into account [121].

According to Table 8, all the CFI, IFI, and TLI values for configural invariance, metric invariance, and scalar invariance were high enough (>0.0900) to be considered an accepted fit [126]. The findings demonstrated that the specified criteria were met by the remaining fit indices. The configuration invariance, metric invariance, and scalar invariance were acceptable. Thus, the full MI was constructed.

Table 9 displays the GOF measures and thresholds for the multigroup structural model. All the results were acceptable because they were above the minimum requirements that were specified. The comparative fit index (CFI; 0.923), incremental fit index (IFI; 0.924), Tucker–Lewis index (TLI; 0.912), and root mean square error of approximation (RMSEA; 0.049) passed the thresholds.

**Figure 3:**
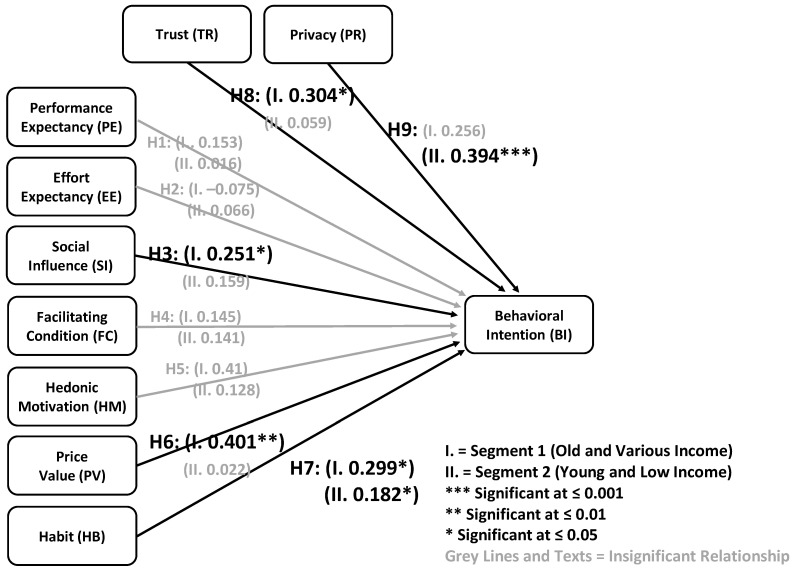
Multigroup structural model. Source: Figure created by authors (2023).

After the MGA was run, the hypothesis test results for Segments 1 (I) and Segment 2 (II) could be analyzed separately. According to Table 10 and Figure 3, H1 (I) and (II), H2 (I) and (II), H3 (II), H4 (I) and (II), H5 (I) and (II), H6 (II), H8 (II), and H9 (I) were not statistically significant, due to the *p*-values of <0.05, <0.01, or <0.001. This insignificant relationship implies that, when separating the data into two segments, performance expectation, effort expectance, facilitating condition, and hedonic motivation did not influence the behavioral intentions to use the platform. This result occurred in both segments. However, H3 (I), H6 (I), H7 (I) and (II), H8 (I), and H9 (II) were accepted because their *p*-values were less than the given level of significance. Additionally, the critical ratio difference revealed a significant path difference only for H7 (critical ratio = |−2.011| > |1.96|), indicating that the loadings of Segments 1 and 2 were statistically different. These results are discussed in more detail in the next section.

## 5. Discussion

Based on the statistical results from the SEM and MGA (Segment 1 vs. Segment 2) analyses, we can discuss the findings from the H1 to H9 tests as follows.

**Hypothesis** **H1.** *Performance expectancy positively influences users’ behavioral intentions to use the circular agricultural waste management platform*.

The findings of the study revealed that the respondents’ performance expectancy had no beneficial effect on their use of the platform (*p*-value = 0.403 > 0.05). This was because “performance expectancy” refers to providing benefits and possibilities that are valuable for users in agricultural waste management, yet most of them did not express a high level of perception of this performance expectancy. Additionally, they may have felt that adopting a platform did not increase their productivity. Consequently, the majority of the respondents did not place a great deal of importance on performance expectancy. The study was not comparable to earlier research by Yeo et al. (2017) [41], who found that performance expectancy was the extent to which users’ propensities to utilize online food-ordering systems were significantly influenced by the usability of the system.

Moreover, based on the results from MGA, the moderation effect of the demographic segments did not affect this relationship. The results revealed that neither Segment 1 (older and various incomes) nor Segment 2 (young and low incomes) moderated the relationship between performance expectancy and behavioral intentions, given the insignificant *p*-values of Segment 1 (*p*-value = 0.288 > 0.05) and Segment 2 (*p*-value = 0.852 > 0.05). In this respect, performance expectancy did not influence the intentions to use an agricultural waste management platform for the users of all ages and income statuses. This was contradictory to the previous findings of Yusof et al. (2019) [98], Bilgihan (2016) [106], and Naruetharadhol et al. (2022) [109].

**Hypothesis** **H2.** *Effort expectancy positively influences users’ behavioral intentions to use a circular agricultural waste management platform*.

In line with findings from earlier research, this study’s findings (*p*-value = 0.857 > 0.05) revealed that the level of expected effort did not have any impact on the platform utilization [9,44], because most of the respondents were unfamiliar with the technology. As a result, they may have found it difficult to utilize this platform, the interactions between the users and this platform may have been confusing, and the functioning was still problematic. Therefore, the respondents were not concerned about the effort expectancy of using the platform.

The MGA result revealed that the moderation effect of the demographic segments did not affect this relationship either. The findings indicated that the association between effort expectancy and behavioral intentions was not moderated by either Segment 1, which comprised individuals with diverse incomes and advanced ages, or Segment 2, which consisted of young individuals with low incomes. This was evidenced by the non-significant *p*-values of Segment 1 (*p*-value = 0.464 > 0.05) and Segment 2 (*p*-value = 0.474 > 0.05). It can be observed that the intention to utilize an agricultural waste management platform was not affected by the level of effort expectancy among the users in both segments. This assertion is in contrast with the earlier technology adoption research conducted by Yusof et al. (2019) [98] and Naruetharadhol et al. (2022) [109].

**Hypothesis** **H3.** *Social influences positively impact behavioral intentions to use the agricultural waste management platform*.

This gives credibility to the positive benefits that social influence has on behavioral intention (*p*-value < 0.001), which have been found in earlier research [21]. The result of this research implied that the information obtained from others around someone adds to their social influence. This influenced the inclination to use the platform more often, because the respondents may have been interested in a platform for disposing of agricultural waste management. This would also be a way for merchants and farmers to make more money and is expected to have a big impact on society.

Based on the MGA result, social influence (H3) appeared to affect the behavioral intentions in Segment 1 (loading = 0.251, *p*-value = 0.042 < 0.05), but was irrelevant for Segment 2 (loading = 0.159, *p*-value = 0.052). This result implies that the users in Segment 1 (older and various incomes) needed to have influences from their peers or third parties prior to actual use, while the users in Segment 2 (young and low incomes) did not need this. Because the older users in Segment 1 were not technology-oriented [98], social influence would play a critical role in attracting their intention.

**Hypothesis** **H4.** *Facilitating conditions positively influence users’ behavioral intentions to use an agricultural waste management platform*.

This conclusion from the structural model revealed that previous research on the acceptability of the platform’s facilitating conditions had a major influence on the users’ intentions, given the *p*-value of 0.03 (<0.05 level of significance). This finding supports Lu et al. (2005) [52] and Naruetharadhol et al. (2023) [9]. However, when strictly analyzing this relationship using a *p*-value of 0.01, this hypothesis can be rejected and would lead to unsupported results for MGA.

However, the result of MGA revealed that the facilitating condition did not affect the userss behavioral intentions in both Segments 1 and 2, given the *p*-values of 0.139 (>0.05) and 0.134 (>0.05), respectively. Most of the respondents believed the platform having customer services to help customers and provide information was not necessary. They also expected that the platform would not be compatible with the other technologies that they used. Hence, the MGA results contradicted Lu et al. (2005) [52] and Naruetharadhol et al. (2023) [9].

**Hypothesis** **H5.** *Hedonic motivation positively influences users’ behavioral intentions to use an agricultural waste management platform*.

The research results showed that the hedonic motivation of the respondents did not impact their intentions to use the platform (*p*-value = 0.227 > 0.05), which differed from previous studies by Venkatesh et al. (2012) [36] and Naruetharadhol et al. (2022) [109], wherein hedonic motivation was shown to be a crucial component of behavioral intentions to adopt mobile internet in a consumer setting.

The MGA result also revealed that demographic segments did not moderate this structural relationship. This was because most of the respondents assumed that the platform was not focused on entertainment or enjoyment enough to affect the users’ intentions. According to the findings, the demographic segments did not have an impact on the relationship between hedonic motivation and behavioral intentions. Specifically, Segment 1, which included individuals with varying income levels and an older age (*p*-value = 0.681 > 0.05), and Segment 2, which was made up of younger individuals with lower incomes (*p*-value = 0.231 > 0.05), did not affect this association. These MGA results are unsupported by Venkatesh et al. (2012) [36] and Naruetharadhol et al. (2022) [109].

**Hypothesis** **H6.** *Price value positively influences users’ behavioral intentions to use an agricultural waste management platform*.

The findings of the study revealed that respondents’ price values had no effect on their use of the platform (*p*-value = 0.126 > 0.05). Users may have been uninterested in the platform because they perceived it to be a relatively new platform (farmers and sellers), making it difficult to generate money. Some responders may also have been concerned about the expenses associated with utilizing this platform. For this reason, this hypothesis had no significant effect compared to previous research [40].

When using the MGA approach, nevertheless, price value (H6) highly influenced the behavioral intentions in Segment 1 (loading = 0.401, *p*-value = 0.005 < 0.001), but not in Segment 2 (loading = 0.022, *p*-value = 0.827). Table 10 also indicates a critical ratio difference of |−2.011|, which is more than the threshold of |1.96|, confirming that the loading of Segment 1 was statistically greater than that of Segment 2. This reveals that the users in Segment 1 tended to pay attention to price value because they expected the platform to be reasonably priced to attract their behavioral intention [59]. On the other hand, the users in Segment 2 were not more likely to be concerned about price value.

**Hypothesis** **H7.** *Habit positively influences users’ behavioral intentions to use an agricultural waste management platform*.

The findings of the SEM were consistent with previous studies by Venkatesh et al. (2012) [36], which discovered that habit affected behavioral intentions (*p*-value < 0.001). This research identified that habit directly affected the behavioral intentions toward using the platform. Due to the platform information given to these potential users before each survey, most respondents are expected to use this platform and the platform is expected become a habituation for users to sell agricultural waste.

Furthermore, when considering the MGA result, habit affected the behavioral intentions in both Segment 1 (loading = 0.299, *p*-value = 0.020 < 0.05) and Segment 2 (loading = 0.182, *p*-value = 0.029 < 0.05). The impact was slightly higher in Segment 1 than in Segment 2. This shows that the users in both segments expected to use the platform often or as a part of their regular activities. This platform would be their first choice when it comes to working.

**Hypothesis** **H8.** *Trust positively influences users’ behavioral intentions to use an agricultural waste management platform*.

The SEM result suggested that trust robustly enhanced the behavioral intentions to use a platform for agricultural waste management (*p*-value = 0.046 < 0.05). According to the survey results, both the farmers and sellers felt that the usage of this platform was dependable, that it maintained its promises to its users, and that the material on this platform was reputable. This is consistent with the earlier literature, which has demonstrated that trust has a noticeable effect on behavioral intentions [37].

The MGA result suggested that trust influenced the behavioral intentions in Segment 1 (loading = 0.304, *p*-value = 0.032 < 0.05), but not in Segment 2 (loading = 0.059, *p*-value = 0.575). The test result demonstrated that trust may have better influenced the users in Segment 1 (older users) than in Segment 2 (younger users).

**Hypothesis** **H9.** *Privacy positively influences users’ behavioral intentions to use an agricultural waste management platform*.

The results indicated that privacy had a great impact on the users’ behavioral intentions to use the platform (*p*-value < 0.001), comparable to prior research from Yuduang et al. (2022) [79]. According to the survey findings, the respondents believed that the privacy of this platform’s users was protected and that the platform would keep the information of the participants safe. These findings are encouraging, because respondents’ perceptions of privacy are a significant factor in their decision to use a platform.

For the MGA result, privacy also did not impact the behavioral intentions in Segment 1 (loading = 0.256, *p*-value = 0.063), but strongly affected them in Segment 2 (loading = 0.394, *p*-value = ***). Because the respondents in Segment 1 were mostly from Generation X and baby boomers, they may not place as much emphasis on data privacy in their use of online platforms as the respondents in Segment 2 do, which was made up of people in the age range with unlimited internet access, combined with the development of technologies and social media.

## 6. Research Implications

Primary stakeholders, such as farmers and retailers, were presented with the research findings. An agricultural platform is an application that acts as the intermediary that helps two parties (farmer/seller and organization) to make a deal through this platform. Thus, the users, involving fruit and vegetable farmers and sellers, can earn more income by selling agricultural and food leftovers through this application. The developers should create the best quality platform features that are user-friendly and facilitating, adaptable to user habits, trustworthy, and privacy-concerned. Additionally, this platform should involve marketing features that enhance its social influence, such as referencing and testimonials, because these can impact user intentions to use the platform. This research has clear benefits for marketing and development teams, who would benefit from having access to and knowledge of user behavior intentions and the user types in specialized markets for selling and buying food waste as a result of this study.

In addition, marketers should utilize different strategies for attracting users in different demographic segments: (1) older users with various income ranges, and (2) younger users with low incomes. Marketers may create advertisements to attract older people with various income ranges to use the platform, focusing on social influence by utilizing peers or celebrities as influencers. More than that, the developer should allow older people with various incomes to set their selling prices, attracting more utilization. The developer should create a platform with features similar to other everyday-use platforms in order to create familiarity with using the platform for both Segments 1 and 2. The developer should establish reliable content, because older people pay attention to reliability. Concerning younger users, the team developer should be concerned about privacy and security to attract their utilization.

## 7. Research Limitations and Future Research

This study contributes significantly to academic and business processes. However, there are still certain limits to this research. The questionnaire was only administered to residents in the northeast of Thailand; hence, the results may not be representative of sample groups in other regions of the country. Data may be collected from other regions of Thailand to ascertain and compare them with the present study’s results.

Future research may adapt the variables of the UTAUT2 by adding more variables to this structural model to understand users’ behavior intentions. These additional variables may include environmental awareness, perceived risk, personal innovativeness in IT, and resistance to change. In addition, it may shift the moderator of the user segment to farmers and sellers, in order to analyze the specific behaviors of these users. Finally, after the actual platform is launched, it is possible to utilize different research designs, for instance, exploring the actual use behavior through a quasi-experimental design with regard to the platform’s features, user interface (UI), and user experience (UX).

## 8. Conclusions

The purpose of this study was to identify the different demographic segments of users, as well as the significant factors that influence the behavioral intentions to utilize a circular-economy-based digital platform for fruit and vegetable waste management, based on the Unified Theory of the Adoption and Utilization of Technology 2 (UTAUT2). The study found two user segments: (1) older and various-income users, and (2) young and low-income users. Our data suggest that social influence, price value, habit, trust, and privacy are essential elements impacting these user behavioral intentions when using a fruit and vegetable waste management platform. Contrary to the UTAUT2, there was no substantial relationship between performance expectancy, effort expectancy, facilitating condition, hedonic incentive, and behavioral intentions towards adopting the technology. The results show that the attitudes and behaviors of surrounding society influence consumers’ adoption of waste management platforms, especially for older and various-income users. When motivated by a group of people who influence beliefs or feelings, such as a colleague or a person who has the same or a similar occupation, the more probable it is that the Segment 1 users would be driven, convinced, and followed by them. Additionally, the results revealed that price value tended to influence the users in Segment 1, but did not affect those in Segment 2. Moreover, the data revealed that consumers wanted to engage in online behavior owing to habit in both segments, as familiarity influenced the propensity to use the platform. Its intended use is equally vital to directing its usage behavior. Therefore, the platform developers should promote the benefits of its use and encourage continuing use by, for example, customer service, a user-friendly and straightforward platform, or more services. Furthermore, the results demonstrated that users have online behavioral intentions due to trust. Explicitly, trust increased interest in using the platform for Segment 1 users. This might establish credibility and influence behavioral intentions. Hence, the platform developers should provide clear information and contact options to increase the platform’s credibility. Additionally, the results demonstrated that users have online behavioral intentions due to privacy, especially the younger users in Segment 2; thus, the platform developers should create the most secure platform possible to safely store user information without publishing it. Therefore, this study suggests that the platform developers should promote users’ behavioral intentions differently through specific target groups for fruit and vegetable sellers and farmers from various age and income groups.

## Figures and Tables

**Table 1:** Descriptive statistics for the demographic profiles.

Demographic Profile	Measure	Segment 1		Segment 2		Total		Chi-Square Test
		*n*	%	*n*	%	*n*	%	
Segment size		122		204		326		
Gender	Male	55	16.9	76	23.3	131	40.2	0.163
	Female	67	20.6	128	39.3	195	59.8	
Age	Baby Boomer	31	9.5	0	0	31	9.5	***
	Gen X	83	25.5	0	0	83	25.5	
	Gen Y	5	1.5	76	23.3	81	24.8	
	Gen Z	3	0.9	128	39.3	131	40.2	
Income (Baht)	Less than 15,000	54	16.6	126	38.7	180	55.2	
	15,001–20,000	23	7.1	57	17.5	80	24.5	***
	20,001–25,000	12	3.7	20	6.1	32	9.8	
	25,001–30,000	15	4.6	1	0.3	16	4.9	
	More than 30,000	18	5.5	0	0	18	5.5	

Source: data adapted from authors (2023). Note: *** denotes significant at <0.01; 1 dollar is roughly 37.90 Bath (Retrieved from: wise.com, visit date 13 October 2022).

**Table 2:** Independent sample *t*-test result.

Psychographic Profile	Measure	Segment 1		Segment 2		Mean Diff	t	*t*-Test
		Mean	SD	Mean	SD			
Performance Expectancy	PE1	3.76	0.834	3.93	0.915	−0.164	−1.620	0.334
	PE2	3.61	0.886	3.87	0.886	−0.253	−2.494	0.078
	PE3	3.60	1.081	3.94	0.886	−0.343	−3.110	***
Effort Expectancy	EE1	3.65	1.012	3.79	0.888	−0.142	−1.323	**
	EE2	3.73	0.927	3.85	0.894	−0.119	−1.142	0.053
	EE3	3.83	0.924	3.92	0.987	−0.089	−0.805	0.483
	EE4	3.75	0.875	3.92	0.961	−0.163	−1.527	0.702
Social Influence	SI1	3.57	1.052	3.76	0.779	−0.186	−1.825	***
	SI2	3.33	1.071	3.72	0.852	−0.388	−3.604	***
	SI3	3.34	0.994	3.77	0.849	−0.425	−4.104	**
	SI4	3.49	0.956	3.72	0.864	−0.224	−2.175	0.057
Facilitating Condition	FC1	3.86	0.930	3.80	0.803	0.062	0.632	**
	FC2	3.57	0.953	3.82	0.811	−0.250	−2.517	***
	FC3	3.88	0.809	3.91	0.960	−0.030	−0.287	0.418
	FC4	3.94	0.865	3.88	0.924	−0.060	0.584	0.512
Hedonic Motivation	HM1	3.79	0.964	3.73	0.877	0.056	0.543	0.101
	HM2	3.35	0.961	3.68	0.890	−0.324	−3.087	0.240
	HM3	3.36	0.954	3.66	0.881	−0.301	−2.894	0.167
	HM4	3.84	0.894	3.80	0.952	0.032	0.302	0.659
Price Value	PV1	4.05	0.952	3.85	0.983	0.201	1.808	0.999
	PV2	3.95	0.935	3.80	0.938	0.152	1.416	0.628
	PV3	4.01	0.895	3.83	0.980	0.180	1.655	0.718
	PV4	3.91	0.927	3.82	0.992	0.086	0.779	0.267
Habit	HB1	3.59	0.985	3.65	0.877	−0.062	−0.587	0.097
	HB2	3.27	1.240	3.58	0.887	−0.313	−2.646	***
	HB3	3.38	1.063	3.69	0.910	−0.309	−2.787	*
Trust	TR1	4.06	0.893	3.87	0.938	0.185	1.752	0.518
	TR2	3.71	0.904	3.81	0.939	−0.101	−0.949	0.810
	TR3	3.86	0.930	3.83	0.927	0.027	0.257	0.649
	TR4	3.97	0.881	3.87	0.919	0.100	0.961	0.418
Privacy	PR1	3.93	0.946	3.89	0.948	0.039	0.359	0.825
	PR2	3.89	0.925	3.81	1.026	0.085	0.747	0.233
	PR3	3.98	0.909	3.83	1.013	0.150	1.346	0.519
	PR4	3.96	0.827	3.83	1.067	0.131	1.159	**
Behavioral Intention	BI1	3.79	0.845	3.84	0.946	−0.051	−0.493	0.905
	BI2	3.51	0.893	3.74	0.955	−0.232	−2.174	0.899
	BI3	3.64	0.863	3.78	0.960	−0.140	−1.323	0.902
	BI4	3.74	0.851	3.79	0.908	−0.056	−0.556	0.954

Source: data adapted from authors (2023). Note: *** denotes significant at <0.001, ** at <0.01, and * at <0.05.

**Table 3:** The goodness of fit of measurement model.

Fit Indices	Value	Threshold	Assessment
*p*-value	≤0.001		Acceptable for complex model
CMIN/*df*	2.655	<3.00	Pass
CFI	0.913	>0.900	Pass
IFI	0.914	>0.900	Pass
TLI	0.900	>0.900	Pass
RMSEA.	0.071	<0.100	Pass

Source: data adapted from authors (2023). Note: CMIN/*df* = Chi-square/degree of freedom, CFI = comparative fit index, IFI = incremental fit index, TLI = Tucker–Lewis index, and RMSEA = root mean square error approximation.

**Table 4:** Convergent validity.

Construct	Indicator	Loading	*p*-Value	Cronbach’s Alphas	AVE	CR
				(Threshold = 0.70)	(Threshold = 0.50)	(Threshold = 0.70)
Performance Expectancy	PE2	0.764	***	0.862	0.685	0.867
	PE3	0.878	***			
	PE4	0.873	***			
Effort Expectancy	EE1	0.77	***	0.907	0.711	0.907
	EE2	0.825	***			
	EE3	0.902	***			
	EE4	0.869	***			
Social Influence	SI1	0.734	***	0.902	0.681	0.895
	SI2	0.842	***			
	SI3	0.878	***			
	SI4	0.839	***			
Facilitating Condition	FC1	0.813	***	0.894	0.695	0.901
	FC2	0.768	***			
	FC3	0.863	***			
	FC4	0.885	***			
Hedonic Motivation	HM1	0.715	***	0.868	0.633	0.873
	HM2	0.869	***			
	HM3	0.828	***			
	HM4	0.764	***			
Price Value	PV1	0.877	***	0.933	0.782	0.935
	PV2	0.914	***			
	PV3	0.905	***			
	PV4	0.839	***			
Habit	HB1	0.822	***	0.897	0.749	0.899
	HB2	0.876	***			
	HB3	0.896	***			
Trust	TR1	0.892	***	0.94	0.799	0.941
	TR2	0.837	***			
	TR3	0.921	***			
	TR4	0.922	***			
Privacy	PR1	0.845	***	0.943	0.807	0.943
	PR2	0.932	***			
	PR3	0.922	***			
	PR4	0.891	***			
Behavioral Intention	BI1	0.893	***	0.94	0.801	0.942
	BI2	0.855	***			
	BI3	0.918	***			
	BI4	0.913	***			

Source: data adapted from authors (2023). Note: AVE = average variance extracted, CR = composite validity, *** significant <0.001.

**Table 5:** Discriminant validity.

**Fornell and Larcker Criterion**										
	**BI**	**PR**	**TR**	**HB**	**PV**	**HM**	**FC**	**SI**	**EE**	**PE**
BI	0.895									
PR	0.685	0.898								
TR	0.644	0.818	0.894							
HB	0.67	0.359	0.487	0.865						
PV	0.667	0.789	0.804	0.389	0.884					
HM	0.733	0.605	0.633	0.66	0.658	0.796				
FC	0.683	0.675	0.706	0.451	0.746	0.654	0.833			
SI	0.718	0.443	0.481	0.718	0.476	0.712	0.535	0.825		
EE	0.638	0.661	0.697	0.45	0.722	0.613	0.713	0.574	0.843	
PE	0.649	0.438	0.513	0.666	0.478	0.617	0.607	0.696	0.633	0.828
**HTMT Ratio Approach**										
	**BI**	**PR**	**TR**	**HB**	**PV**	**HM**	**FC**	**SI**	**EE**	**PE**
BI	-	-	-	-	-	-	-	-	-	-
PR	0.685	-	-	-	-	-	-	-	-	-
TR	0.644	0.819	-	-	-	-	-	-	-	-
HB	0.617	0.359	0.487	-	-	-	-	-	-	-
PV	0.667	0.789	0.804	0.390	-	-	-	-	-	-
HM	0.734	0.606	0.634	0.661	0.659	-	-	-	-	-
FC	0.683	0.675	0.707	0.451	0.746	0.739	-	-	-	-
SI	0.708	0.437	0.474	0.707	0.469	0.702	0.528	-	-	-
EE	0.638	0.662	0.698	0.451	0.722	0.613	0.713	0.566	-	-
PE	0.650	0.438	0.513	0.667	0.478	0.618	0.607	0.687	0.634	-

Source: data adapted from authors (2023). Note: BI = Behavioral Intention, PR = Privacy, TR = Trust, HB = Habit, PV = Price Value, HM = Hedonic Motivation, FC = Facilitating Condition, SI = Social Influence, EE = Effort Expectancy, and PE = Performance Expectancy.

**Table 6:** The goodness of fit of structural model.

Fit Indices	Value	Threshold	Assessment
*p*-value	≤0.001		Acceptable for complex model
CMIN/df	2.470	<3.00	Pass
CFI	0.925	>0.900	Pass
IFI	0.926	>0.900	Pass
TLI	0.915	>0.900	Pass
RMSE	0.067	<0.100	Pass

Source: data adapted from authors (2023). Note: CMIN/*df* = Chi-square/degree of freedom, CFI = comparative fit index, IFI = incremental fit index, TLI = Tucker–Lewis index, RMSEA = root mean square error approximation.

**Table 7:** Hypothesis test results from.

Hypothesis	Endogenous Variable	Exogenous Variable	Standardized Estimate	*p*-Value	Result
H1	Performance Expectancy	Behavioral Intention	0.054	0.403	Rejected
H2	Effort Expectancy	Behavioral Intention	−0.012	0.857	Rejected
H3	Social Influence	Behavioral Intention	0.222	***	Supported
H4	Facilitating Condition	Behavioral Intention	0.143	0.030 **	Supported
H5	Hedonic Motivation	Behavioral Intention	0.085	0.227	Rejected
H6	Price Value	Behavioral Intention	0.119	0.126	Rejected
H7	Habit	Behavioral Intention	0.259	***	Supported
H8	Trust	Behavioral Intention	0.158	0.046 **	Supported
H9	Privacy	Behavioral Intention	0.365	***	Supported

Source: data adapted from authors (2023). Note: *** denotes significant at ≤0.001 and ** at ≤0.05.

**Table 8:** Measurement invariance.

Fit Indices	Configural Invariance	Metric Invariance	Scalar Invariance	Threshold
*p*-value	≤0.001	≤0.001	≤0.001	
CFI	0.923	0.917	0.906	>0.900
IFI	0.924	0.918	0.907	>0.900
TLI	0.912	0.917	0.906	>0.900
RMSEA	0.049	0.051	0.053	<0.10
	Acceptable	Acceptable	Acceptable	

Source: data adapted from authors (2023). Note: CFI = comparative fit index, IFI = incremental fit index, TLI = Tucker–Lewis index, RMSEA = root mean square error approximation.

**Table 9:** The goodness of fit of the multigroup structural model.

Fit Indices	Value	Threshold	Assessment
*p*-value	≤0.001		Acceptable for complex model
CMIN/*df*	1.785	<3.00	Pass
CFI	0.923	>0.900	Pass
IFI	0.924	>0.900	Pass
TLI	0.912	>0.900	Pass
RMSE	0.049	<0.100	Pass

Source: data adapted from authors (2023). Note: CMIN/*df* = Chi-square/degree of freedom, CFI = comparative fit index, IFI = incremental fit index, TLI = Tucker–Lewis index, RMSEA = root mean square error approximation.

**Table 10:** Test result of loading differences.

Hypothesis	Relationship	Segment 1			Segment 2			Critical Ratio Difference	Threshold
		Std. Est.	*p*-Value	Result	Std. Est.	*p*-Value	Result		
H1	PE → BI	0.153	0.288	Rejected	0.016	0.852	Rejected	−0.939	|1.96|
H2	EE → BI	−0.075	0.464	Rejected	0.066	0.474	Rejected	1.024	|1.96|
H3	SI → BI	0.251	0.042 *	Supported	0.159	0.052	Rejected	−0.34	|1.96|
H4	FC → BI	0.145	0.139	Rejected	0.141	0.134	Rejected	0.172	|1.96|
H5	HM → BI	0.041	0.681	Rejected	0.128	0.213	Rejected	0.347	|1.96|
H6	PV → BI	0.401	0.005 **	Supported	0.022	0.827	Rejected	−2.011 *	|1.96|
H7	HB → BI	0.299	0.020 *	Supported	0.182	0.029 *	Supported	−0.348	|1.96|
H8	TR → BI	0.304	0.032 *	Supported	0.059	0.575	Rejected	1.224	|1.96|
H9	PR → BI	0.246	0.063	Rejected	0.394	***	Supported	1.528	|1.96|

Source: data adapted from authors (2023). Note: *** denotes significant at ≤0.001, ** at ≤0.01, and * at ≤0.05. BI = Behavioral Intention, PR = Privacy, TR = Trust, HB = Habit, PV = Price Value, HM = Hedonic Motivation, FC = Facilitating Condition, SI = Social Influence, EE = Effort Expectancy, and PE = Performance Expectancy.

## Data Availability

Data is contained within the article.

## Appendix A

### Appendix A.1. Questionnaire—Demographic Questions

(1) Gender: a. Male b. Female
(2) Age: a. Gen Z (20–27 years old) b. Gen Y (28–41 years old) c. Gen X (42–56 years old) d. Baby Boomer (57 years old and above)
(3) Income: a. Less than BHT 15,000. b. BHT 15,000–20,000. c. BHT 25,001–30,000. d. More than BHT 30,000.

**Table A1:** Answers in sub-questions are on a 1 to 5 Likert scale; 1 as strongly disagree and 5 as strongly agree.

Constructs	Items	Measurement	Reference
Performance Expectancy (PE)	PE1	I expect that using this platform will increase my chances of success at work, which is important to me.	[36]
	PE2	I expect using this platform will help me accomplish tasks more quickly.	[36]
	PE3	I expect using this platform will increase my productivity.	[36]
Effort Expectancy (EE)	EE1	I expect learning how to use this platform will be easy.	[36]
	EE2	I expect that my interaction with this platform will be clear and understandable.	[36]
	EE3	I expect this platform to be easy to use.	[36]
	EE4	I expect it will be easy for me to become skillful at using this platform.	[36]
Social Influence (SI)	SI1	People who matter to me suggest that I should use this platform.	[36]
	SI2	People who influence my behavior think that I should use this platform.	[36]
	SI3	People whom I respect desire that I employ this platform in agriculture production.	[36]
	SI4	People who use this platform have more prestige in my society.	[36]
Facilitating Condition (FC)	FC1	I expect to have the resources necessary to use this platform.	[36]
	FC2	I expect to have the knowledge necessary to use this platform.	[36]
	FC3	I expect that this platform will be compatible with other technologies I use.	[36]
	FC4	I expect that I can get help from others when I have difficulties using this platform.	[36]
Hedonic Motivation (HM)	HM1	I expect that using this platform is interesting.	[36]
	HM2	I expect that using this platform is enjoyable.	[36]
	HM3	I expect that using this platform is entertaining.	[36]
	HM4	I expect that using this platform is pleasurable.	[21]
Price Value (PV)	PV1	I expect that this platform will be reasonably priced.	[36]
	PV2	I expect that this platform is good value for the money.	[36]
	PV3	I expect that at the current price, this platform provides good value.	[36]
	PV4	I expect this platform fee does not significantly affect my monthly bill for services.	[36]
Habit (HB)	HB1	I expect that using this platform has become a habit for me.	[127]
	HB2	I am addicted to using this platform.	[21]
	HB3	I expect using this platform will be a regular activity for me.	[127]
Trust (TR)	TR1	I believe that using this platform is trustworthy.	[36]
	TR2	I believe that this platform can fulfill its task.	[36]
	TR3	I believe that this platform keeps its promises to its users.	[36]
	TR4	I believe that the content of this platform is reliable.	[21]
Privacy (PR)	PR1	I believe that the privacy of users of this platform is protected.	[21]
	PR2	I believe that personal information stored in this platform system is secure.	[21]
	PR3	I believe that this platform keeps participants’ information secure.	[21]
	PR4	I believe this platform does not use GPS or track mobile phone locations after use.	[21]
Behavioral Intention (BI)	BI1	I am interested and expect to use this platform in the future.	[36]
	BI2	I will always try to use this platform in daily life.	[36]
	BI3	I plan to use this platform in the future.	[36]
	BI4	I predict I will use this platform in the future.	[17]
